# Spontaneous Demonstration Of Counterclockwise Right Atrial Activation Following Successful Isthmus Ablation

**Published:** 2011-11-15

**Authors:** Vivek Chaturvedi, Neeraj Parakh, Vijay Trehan, Sanjay Tyagi

**Affiliations:** Department of Cardiology, G. B. Pant Hospital, New Delhi, India

**Keywords:** Atrial flutter, cavo-tricuspid isthmus, sinus node dysfunction

## Abstract

We describe an uncommon case of typical flutter with symptomatic sinus node dysfunction, in which a permanent junctional rhythm developed following ablation of the cavo-tricuspid isthmus. This rhythm activated the right atrium in counter clockwise manner thus providing spontaneous proof of unidirectional isthmus block, a phenomenon that is usually demonstrated by proximal coronary sinus pacing.

## Case report

**Figure 1:**
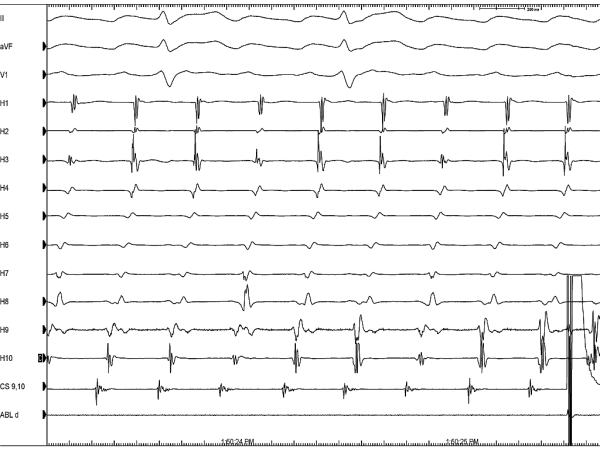
Counterclockwise atrial flutter at baseline

**Figure 2:**
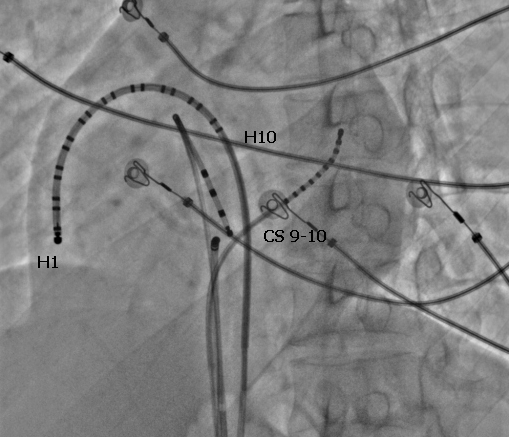
Fluoroscopic image of the catheter ablation of isthmus-dependent atrial flutter

**Figure 3:**
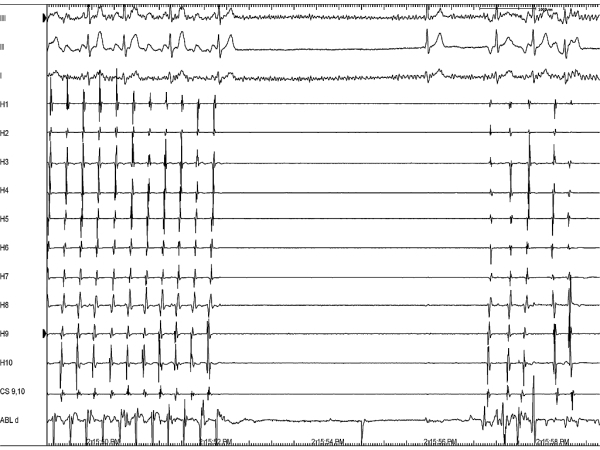
Sinus node arrest after atrial flutter ablation

**Figure 4:**
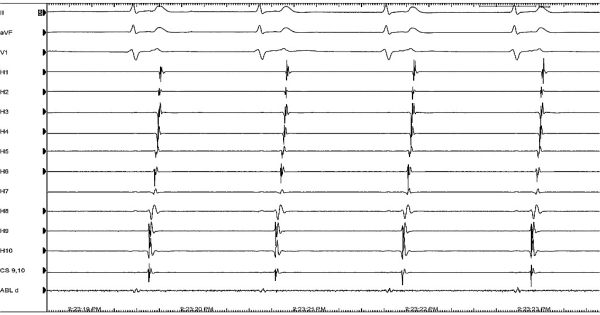
Spontaneous counterclockwise activation of right atrium after ablation

A 24 year old man presented with history of shortness of breath, paroxysmal palpitations and one episode of syncope in the recent past. His baseline electrocardiogram showed typical atrial flutter with variable ventricular rate and the holter revealed presence of persistent atrial flutter with occasional termination followed by prolonged sinus pauses. On echocardiography, he had presence of mild left ventricular dysfunction but there were no other structural abnormalities. He underwent an electrophysiology study, which revealed ongoing isthmus dependent counterclockwise atrial flutter (Figure 1) at baseline and the diagnosis was further confirmed by entrainment. During radiofrequency ablation (Figure 2 shows a duo-decapolar catheter with distal electrode H1 in the lower lateral right atrium and proximal electrode H10 at the inter-atrial septum, a decapolar catheter in coronary sinus, a quadripolar catheter in right ventricular apex, and an ablation catheter on the cavo-tricuspid isthmus), his flutter terminated following which there was a sinus node arrest and a junctional rhythm ensued (Figure 3). This junctional rhythm activated the right atrium in a counterclockwise manner with the lower lateral right atrium getting activated last (Figure 4). This is a spontaneous demonstration of what is usually done as a manoeuvre to establish conductional block across the isthmus namely, pacing of right atrium from proximal coronary sinus or right ventricle and observing the activation pattern.

The ablation was continued with atrial pacing. At the end of procedure he continued to have a stable junctional rhythm with no provocable atrial flutter and bidirectional block of the isthmus. He subsequently required permanent pacemaker implantation, after few days of observation failed to show resolution of the sinus node arrest and an unstable junctional rhythm with intermittent requirement of pacing.

